# Evaluating mechanisms of change in an oral hygiene improvement trial with older adults

**DOI:** 10.1186/s12903-021-01701-1

**Published:** 2021-07-21

**Authors:** Jean Schensul, Susan Reisine, Apoorva Salvi, Toan Ha, James Grady, Jianghong Li

**Affiliations:** 1 Institute for Community Research, 2 Hartford Square West, St. 100, Hartford, CT 06117 USA; 2 University of Connecticut School of Dental Medicine, 263 Farmington Avenue, Farmington, CT 06107 USA; 3 University of Pittsburgh Medical Center, 2118 Public Health, 130 DeSoto Street, Pittsburgh, PA 15261 USA; 4 University of Connecticut School of Medicine, 195 Farmington Avenue, Farmington, CT 06107 USA; 5 Department of Emergency Medicine, School of Medicine, Oregon Health and Science University, Portland, OR 97239 USA

**Keywords:** Oral health, Older adults, Clinical trial, Prevention, Disparities, Multilevel, Counseling, Campaign, Intervention mechanisms

## Abstract

**Background:**

This paper compares the relationship between theoretically-driven mechanisms of change and clinical outcomes across two different interventions to improve oral hygiene of older adults participating in a group randomized trial.

**Methods:**

Six low-income senior residences were paired and randomized into two groups. The first received a face to face counseling intervention (AMI) and the second, a peer-facilitated health campaign (three oral health fairs). Both were based on Fishbein’s Integrated Model. 331 participants were recruited at baseline and 306 completed the post-assessment one month after intervention. Clinical outcomes were Gingival Index (GI) and Plaque score (PS), collected by calibrated dental hygienists. Surveys obtained data on patient background characteristics and ten mechanisms of change including oral health beliefs, attitudes, norms and behaviors. GLMM was used to assess the effects of time, intervention arm, participant characteristics, intervention mechanisms and differences between the two interventions over time in relation to outcomes.

**Results:**

At baseline, both groups had similar background characteristics. Both groups improved significantly in outcomes. Overall GI scores changed from baseline mean of 0.38 (SD = .032) to .26 (SD = .025) and PS scores changed from baseline mean of 71.4 (SD = 18%) to 59.1% (SD = 21%). T-tests showed that fears of oral disease, oral health intentionality, oral health norms, worries about self-management of oral health, flossing frequency and sugar control improved significantly in both interventions from baseline to post intervention. Oral health self-efficacy, perceived risk of oral health problems, oral health locus of control and brushing frequency improved significantly only in the counseling intervention. GLMM models showed that the significant predictors of GI improvement were intentionality to perform oral hygiene, locus of control, and improvement in frequency of brushing and flossing in association with the counseling intervention. Predictors of PS improvement were worries about oral hygiene self-management and fear of oral diseases, in association with the counseling intervention. In the reduced final models, only oral health locus of control (predicting GI) and fears of oral diseases (predicting PS) were significant in association with the counseling intervention. Locus of control, a key concept in oral hygiene interventions including the IM was the main contributing mechanism for GI improvement. Fear, an emotional response, drove improvement in PS, reinforcing the importance of cognitive/emotional mechanisms in oral hygiene interventions.

**Conclusions:**

Though both groups improved in outcomes, GI and PS outcomes improved more in response to the counseling intervention than the campaign. The counseling intervention had an impact on more mechanisms of change than the campaign. Improvements in intervention mechanisms across both interventions however, suggest a closer examination of the campaign intervention impact on outcomes over time.

*Trial Registration*: Clinicaltrials.gov NCT02419144, first posted April 17, 2015.

## Background

Oral health is critical for good general health. The World Health Organization [1] and the Surgeon General of the United States [2, 3], have called for improving access to oral health treatment and to preventive public health practices. Older adults experience more disparities in both [4, 5]. Some reasons include limited access to quality dental treatment, and few opportunities to experience preventive instruction that would help to prevent caries, periodontal disease and edentulism [6].

There is not yet consensus on what improves oral hygiene, especially in racially, ethnically, linguistically and culturally diverse low-income older populations. Some general reviews support behavioral interventions [7]; others suggest that knowledge, and self-efficacy are critical to achieve successful outcomes [8]. Further, there are few detailed descriptions of the components of approaches that show success. Thus, additional exploration of motivational and behavioral factors influencing oral health and hygiene practices and how they are operationalized as mechanisms of change in oral hygiene interventions is called for.

Many oral health researchers argue that improved theory will identify mechanisms that drive interventions making them more subject to empirical testing [9, 10]. Recent reviews of oral health interventions suggest that not one, but a number of domains may be important in shaping health outcomes [11, 12]. The Integrated Model (IM) of health behavior change [13], an approach that incorporates a large number of social, cognitive and behavioral domains, has been shown to be effective in other health related areas [14] and is readily adapted for use in oral health behavior change interventions [15]. Fishbein and colleagues address behavior change mechanisms at multiple levels by including social norms and contextual factors in the Integrated Model. They also identify intentionality as the key motivator, bridging other cognitive and behavioral domains leading to behavior change. The significance of intentionality has been questioned by both theoreticians and interventionists, calling for further examination of its role in health-related cognitive-behavioral models [16, 17].

**Fig. 1:**
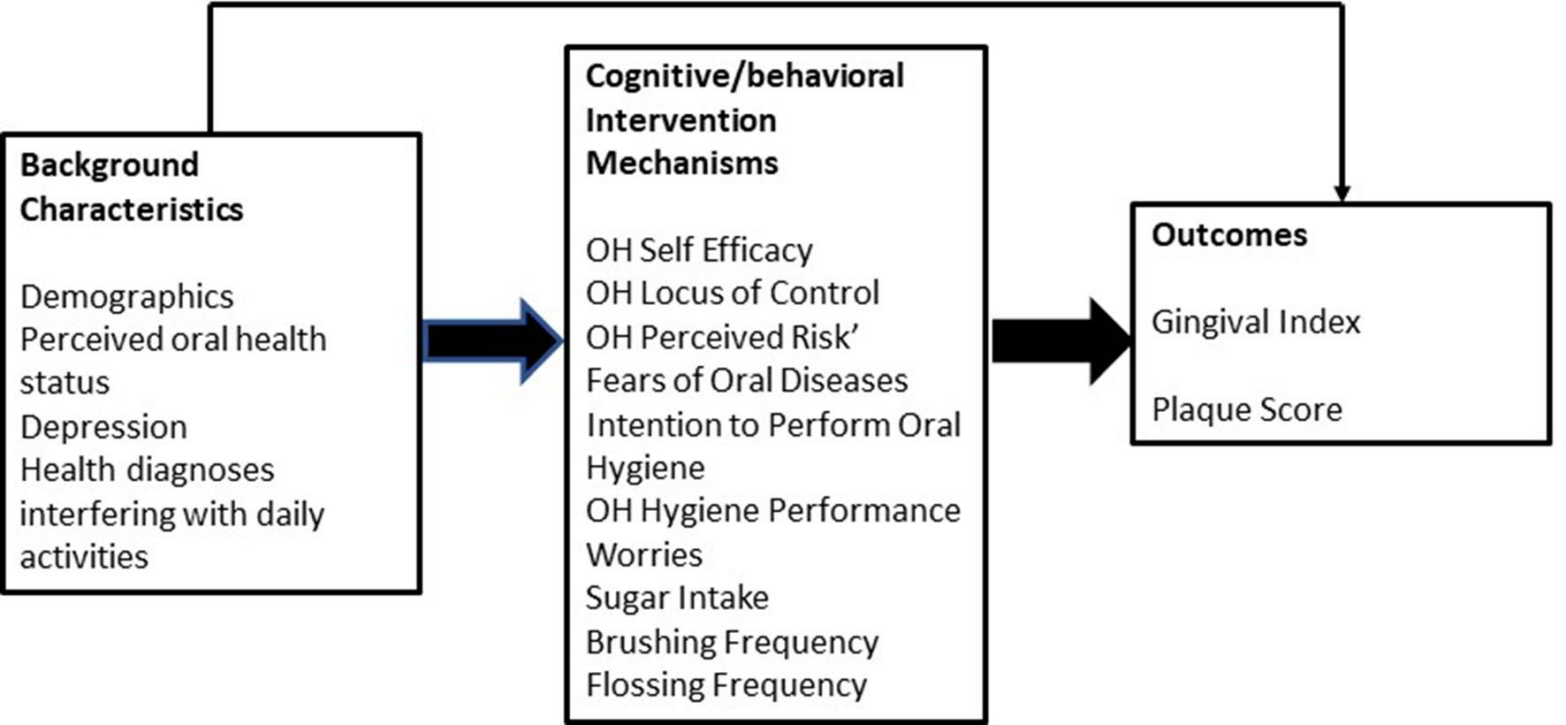
Integrated model of behavior change adapted for oral health

The IM model adapted for oral health behavior change (Fig. 1), illustrates the cognitive/emotional and behavioral domains we have identified as key mechanisms likely to be important in influencing positive oral health clinical outcomes based on the literature and formative research—social norms, beliefs (oral health attitudes, self-efficacy, locus of control), oral health intentions and behaviors (sugar consumption, appropriate tooth brushing and appropriate flossing).

Baseline data from our own clinical trial tested the adapted Fishbein model were used to examine the relationship between these mechanisms of change and clinical outcomes. The results showed that intervention mechanisms functioned differently in relation to the two clinical outcomes, gingival index and plaque score. In the baseline analysis, the mechanisms influencing GI were oral health intentionality, oral health locus of control, and more frequent tooth brushing and flossing. The mechanisms influencing PS were fear of oral diseases, oral health locus of control, and worries about managing oral hygiene. While locus of control was common to both, the differences in mechanisms leading to the two outcomes were notable. At baseline, self-direction and behavioral control of oral health predicted better GI scores while greater fear of oral diseases lower sense of control and more worries about health self-management predicted higher (poorer) plaque scores [18].

To address recent literature supporting the importance of multilevel interventions in confronting complex public health problems [19, 20], and to evaluate the results of the baseline analyses, the mechanisms included in the model were operationalized into 10 intervention domains and implemented through two different interventions: an individual counseling approach delivered one on one through Adapted Motivational Interviewing (referred to as the AMI); and a group norms change oral health campaign consisting of three oral health fairs delivered by peers to all building residents.

In this clinical trial, two cycle crossover design, in the first cycle one group of 3 buildings received the face-to-face counseling intervention (AMI) and the second group of 3 buildings received the peer led campaign. In the next cycle, each building group received the other intervention. The purpose of the first cycle of the study was to compare one intervention against the other to answer whether the interventions had similar or different outcomes, and to examine possible differences in the predictive role of the mechanisms with respect to each intervention. The purpose of the second cycle was to compare the interactive effects of the two different sequences. This paper concentrates on the first cycle, examining differences between the two interventions in outcomes and mechanisms.

The paper addresses several key questions: (a) did the two interventions result in improvements in the clinical outcomes? (b) Did the intervention mechanisms identified and operationalized based on the adapted IM conceptual model change in response to each or both of the interventions? (c) Which mechanisms had the greatest impact on the clinical outcomes in each of the two interventions? The study hypothesized that overall the AMI would achieve better outcomes than the campaign because it was intensive, involved multi-model communication around oral hygiene behavior, and was tailored to individuals with explicit attention to the mechanisms. In contrast the campaign, a tailored group intervention, offered overview exposures to the same intervention mechanisms. At the same time, the study design also provided an opportunity to explore how each intervention affected mechanisms of change which in turn had an impact on outcomes.

## Methods

### Study recruitment and enrolment

Six large rent- subsidized senior apartment buildings in central Connecticut of 125 to 250 apartments were grouped into pairs matched by size and randomized by the study’s biostatistician into two groups of three buildings each. A sample size of 123 per group provided > 90% power to detect mean differences of 0.25 for GI and 0.66 for PS based on data and SDs from a pilot study based on the same protocol in a similar building [21], using a 2-group t-test with a two-sided alpha of 0.05. An attrition rate of 10% was also assumed. Residents of senior housing included older adults, aged 62 and above, and people with disabilities under the age of 62 most of whom were aged 50 and above. *Inclusion criteria were*: ≥ 18 years of age, two teeth or more, no conservator; ability to respond correctly to three of five questions about the study and their rights during the consenting process *Exclusion criteria were*: temporary building resident; under conservatorship; inability to respond correctly to more than three questions about the study and their rights during the consenting process; edentulous; history of infective endocarditis, past six months prosthetic cardiac valve replacement, past six-week myocardial infarction or arterial stent insertion; on dialysis. Eligible candidates signed an informed consent form and completed a baseline (T0) and post intervention (T1) survey and clinical assessment, as well as the intervention assigned to their group. The T1 assessments were administered approximately one month after completion of each intervention. The study recruited and enrolled 331 participants from these buildings from 2015 to 2017, 175 into the AMI counseling intervention and 157 into the campaign intervention. Of these, 165 completed the AMI and 76 unduplicated participants attended at least one of the three campaign events based on sign-in registration at the event. Three hundred and six participants completed the T1 assessment.

### Ethical Approval and Consent to Participate

The study was reviewed annually by the University of Connecticut Health Center IRB and by NIDCR. All procedures were performed in accordance with the US HHS Belmont Report, 1991, and the Revised Common Rule, 2018 and with requirements of the revised the annually approved study protocol.

### Intervention approaches

Intervention activities and processes in both interventions addressed each of the cognitive/emotional and behavioral mechanisms of intervention in the adapted IM model.

1. *The face to face adapted motivational interviewing intervention (AMI:* This 45–60 min counseling approach was administered by trained bilingual English/Spanish speaking oral health educators no more than one month after the baseline survey. It was guided by the IM model. To prepare for the counseling intervention administration, study PIs established cutoff points for scale means in the pre-intervention survey below which it was determined that participants needed intervention [22]. Interventionists followed an intervention protocol that began with cognitive/emotional mechanisms, followed by a behavioral intervention instructional component. First, participants were asked to describe their oral health concerns which the interventionist matched with the mechanisms that scored under the cutoff. Next interventionists discussed with participants the mechanisms scoring under the cutoffs and both engaged in a conversation to determine how to best address them, helped by an explanatory script for each mechanism. Next interventionists proceeded to the behavioral instruction component of the intervention. First, they reviewed with the participants the pre-intervention plaque scoring record which illustrated in red those teeth had plaque accumulations. This record helped the participant to visualize where to target more effective brushing. The second step involved showing two brief videos in English or Spanish to demonstrate correct brushing and flossing techniques. Then participants practiced brushing and flossing on a typodont (model) and were scored and provided with feedback until they had mastered these activities to the best of their ability. This process was referred to as “practice to mastery” (PM). Finally, with the interventionist, participants created their own plan for oral health improvement with strategies for improving brushing, flossing and selected mechanisms, and kept a copy for themselves. All but one enrollee completed the AMI.

2. *The oral health campaign intervention* consisted of three oral health fairs held three to four weeks apart, facilitated by a committee of trained peer volunteers in each building with intervention team support. Bilingual campaign committees consisted of 10–12 volunteers who did not meet study eligibility criteria and represented a diverse cross-section of residents. The volunteer training program began immediately after the baseline assessment was completed for the entire study sample in each building. It consisted of 12 sessions completed over six to eight weeks [22]. Session topics included defining a campaign, team building, oral health and hygiene, and knowledge about all intervention domains (mechanisms of change) in the IM model and their relationship to the desired clinical outcomes. Once completing these basic instructional sessions, volunteers, with staff support, developed messages for residents based on each of the intervention mechanisms, along with interactive games and other activities. Finally, they prepared recruitment strategies and a plan for implementing each of the fairs. Most committee members remained with the campaign for all three oral health fairs. The entire training protocol is posted on the study website (http://www.projectgoh.com).

Fairs were conducted in English and Spanish simultaneously. The protocol for each fair included a standard presentation on oral hygiene by dental hygienists delivered in English and Spanish, followed by a question/answer period. Campaign Committee members assisted by project personnel staffed twelve tables, each with a different message associated with a specific mechanism along with related games and informational handouts. Attendees rotated from table to table querying Committee members. At one of the tables, they were instructed on brushing and flossing using a typodont (model). Both enrolled study participants and non-enrolled residents were welcome at the fairs. Attendance was recorded on a sign-in sheet. Each attendee recorded their visit to each table, evaluating their experience with a “passport”. Their assignment was to complete the passport before leaving the fair. The passport was turned into project staff on departure from each fair. It served as a record of attendance and provided one measure of dosage. All fairs included a raffle and refreshments. Attendance at each fair averaged 45 people including enrolled participants and visitors and a number of attendees attended 2 or all three fairs. Of enrolled participants (n = 157), 76 attended at least one fair. Among the anecdotally derived reasons for non-attendance were disability, depression, other obligations (doctor appointments, work schedule), preference for avoiding public events in their building and the incorrect perception that the fair would be conducted in a language they did not understand.

### Measures

Most cognitive/emotional domains in the IM model (see Fig. 1 above) were adapted from pre-existing literature on factors shown to be associated with oral hygiene behavior. Several scales were based on formative research or pilot testing with the study population including fear of oral diseases and worries about oral health self-management. The latter has been validated and published [23].

*Baseline covariates* included demographics (age < 61 vs 62 and older, gender, income < $900.00 or ≥ $900, ethnicity (Black non-Hispanic, Hispanic, White non-Hispanic plus other) perceived oral health status rated on a four-point Likert scale as poor (1), fair (2), good (3) or excellent (4), and treated as both a continuous and categorical variable, dichotomized as poor/fair vs good/excellent [24], number of diagnoses that interfered with daily activities (0 and 1or more) and depression measured with the CES-D short form (≥ 4 high versus < 4 low) [25].

*Cognitive/emotional Mechanisms* included:

1. *Oral health self-efficacy*: 5 items (α .603) with responses as 4-point Likert scales ranging from strongly agree (4) to strongly disagree (1) [26];
2. Oral health *locus of control*: seven items (α .72) with responses as 4-point Likert scales 1 (low)–4 (high) for both scales [26];
3. *perceived oral health risks* (chances of getting specific health problems associated with oral health) [22]: five questions with responses rated 1–4 with 4 as least chances (α .76);
4. *Fears of oral diseases* [22]: 4 items rated 1–4, with 4 as no fear (α .82);
5. *Intentionality to perform oral hygiene* [27]: a 6-item scale with responses rated as 0 (no intention) to 2 (high intention) (α 72);
6. *Importance of oral health behavior* [28]^.^ 9 items rated from 1 not important to 5 important (α .672.);
7. *oral health self-management worries scale* (OHWSMS): 19 questions with responses rated 1–4 with 4 as least worried (α .93) [23].

*Behavioral mechanisms* included:

1. *sugar intake*: five questions asking about frequency of consumption of sugar and starch (0 is never to 4 as > five times a day);
2. *brushing frequency*: (1 =  < 2/day, 2 = 2 + /day);
3. *flossing frequency*: (0 =  < 1/day v. 1 = 1 + per day).

*Outcome measures* consisted of the Gingival Index (GI) [29] and Plaque Score (PS) [30], both assessed by two trained dental hygienists calibrated each year against an experienced dental examiner. The GI assessed the status of gingiva associated with 6 surfaces of each tooth, three buccal and three lingual, by scoring for gingival inflammation from 0 = no visible inflammation to 3 = overt inflammation and spontaneous bleeding. For GI, the individual scores and the index mean were calculated by summing all surface GI scores and dividing by the total number of surfaces. To obtain the plaque score, the examining hygienist applied a red disclosing to the teeth and identified and recorded dichotomous presence or absence scores for bacterial plaque on each of 6 tooth surfaces. PS is expressed as a percentage of surfaces stained red with plaque over total number of surfaces, or a ratio. Reliability of the clinical assessments was assessed prior to T0 and T1. Two hygienists conducted the clinical assessments with the dental director of the study as the gold standard. After training and prior to T1, Kappa improved from a difference of 0.45 to 0.54 to a difference of 0.72 to 1.00 for the Gingival Index and from a difference of 0.46 to 0,78 to a difference of 0.77 to 0.94 for plaque scores. Measures and calibration procedures are further described elsewhere [22].

Fidelity measures for the AMI counseling intervention included a record of domains covered in each administration, whether the prepared script was utilized, duration of intervention, record of brushing and flossing skills, and a documented plan, These were reviewed for completeness and accuracy by PIs during each cycle along with reviewing 10% of audio recordings of AMI administrations in English and Spanish. All participant files included recorded plans. Fidelity measures for the oral health fairs included a record of slide presentations at campaign events, attendance via registration plus passport record of attendance, exposure to each message table, and observations of each campaign.

### Statistical analysis

**Fig. 2:**
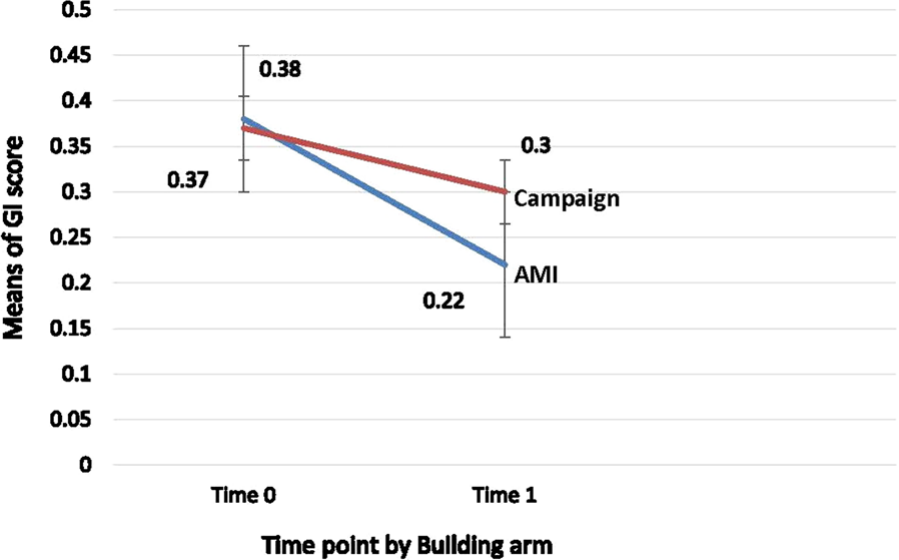
Gingival Index Score mean change from baseline to post intervention (T0 to T1). This figure refers to the difference between the gingival index for the two interventions, AMI counseling and campaign, from baseline (Time 0 to Time 1), approximately 1 month past the intervention. The GI mean dropped in both interventions but it dropped significantly more in the AMI counseling intervention than the campaign

**Fig. 3:**
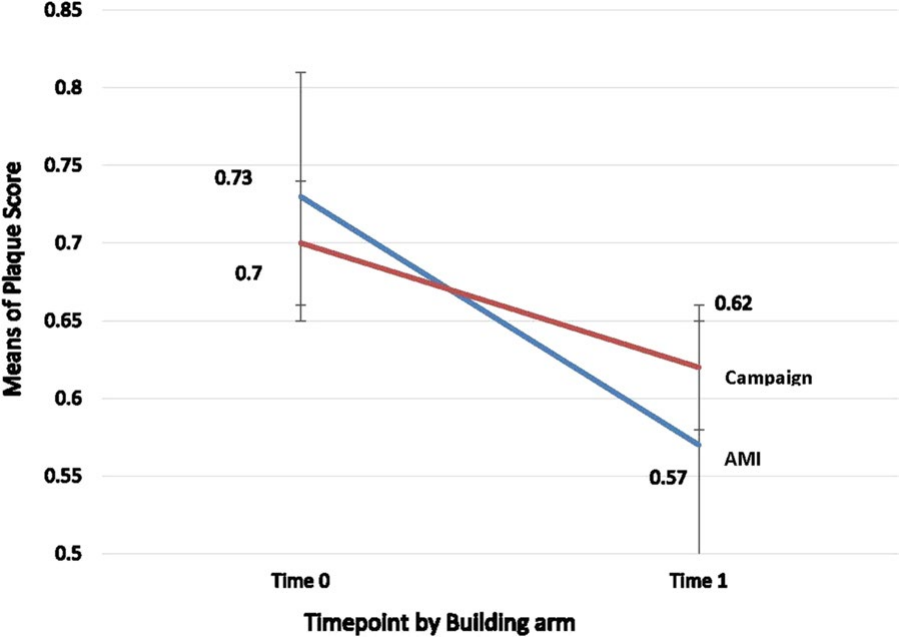
Plaque score mean change from baseline to post intervention (T0 to T1). This figure refers to the difference between the plaque score mean for the two interventions, AMI counseling and campaign, from baseline (Time 0 to Time 1), approximately 1 month past the intervention. The GI mean dropped in both interventions but it dropped significantly more in the AMI counseling intervention than the campaign

To investigate within-group change separately by intervention group, paired *t*-tests are reported for clinical outcomes GI and PS (Table 1) and for intervention mechanisms (Table 2). These tests analyze changes from baseline, but are not meant for comparing the two interventions. To make inferential, adjusted assessments of the intervention, we used repeated measures generalized linear mixed models (GLMMs) with main effects of time (0 vs 1), intervention (AMI vs. Campaign), the (time × intervention) interaction plus covariates of interest (e.g., demographics and health status variables). The interaction terms assess the extent to which the outcomes differ between groups and across time, and interpretations for significant interactions are provided for GI and PS (See Table 4, and Figs. 2 and 3). A final reduced model was fit for including significant mediators, moderators and main effects plus interaction for both GI and PS (Table 5). These GLMM models were estimated in the MIXED procedure in SAS version 9.5. For binary outcomes (brushing and flossing) the general estimating equations (GEE) approach was used in the GENMOD procedure in SAS [31]. A two-sided level of significance of 0.05 was used to determine statistical significance.

**Table 1:** Background characteristics overall and by intervention

Characteristic	Overall percent (n = 331)	Percent AMI counseling intervention (n = 174)	Percent campaign intervention (n = 157)
Gender			
Male	42.0	42.5	41.4
Female	58.0	57.5	58.6
Age (mean = 66.2; SD = 10.4)			
< 62	31.1	31.0	31.2
≥ 62	68.9	68.9	68.8
Race/ethnicity			
Hispanic	58.3	66.1	21.0*
Black Not Hispanic	23.0	24.7	49.7
White Not Hispanic and others	18.7	9.2	29.3
Education			
Less than high school	47.7	49.4	45.7
More than high school	52.3	50.6	54.2
Marital status			
Living alone	83.9	89.1	78.4
Married/with partner	16.0	10.9	21.6
Income (Mean = $1018; SD = $531.6)			
< $900	51.7	45.4	38.8
≥ $900	48.3	54.6	61.2
Time since last visit to the dentist			
< 1 year	55.2	55.5	57.2
≥ 1 year	43.7	44.5	42.8
No. Illness Diagnoses (Mean = 3.8 (SD = 1.4); median = 4			
0–4	67.4	67.8	66.9
4+	32.6	32.2	33.1
No. Diagnoses that Interfere with daily activities (Mean = 1.4 (SD = 1.0); Median = 0)			
0	42.0	43.1	40.8
1+	58.0	56.9	59.2
CES-D-SF (Mean = 4.1 (SD = 2.2); Median = 4			
0–4	57.4	43.1	57.3
4+	42.6	56.9	42.7
Self-Rating of oral health			
Poor and Fair	63.1	60.9	65.6
Good and Excellent	36.9	39.1	34.4

**Table 2:** Mean differences over time in intervention mechanisms for each intervention

Mechanism		AMI counseling intervention				Oral health campaign		
Characteristics (N = 163)		Mean (SD)	Difference in mean (SD)	Paired t-test *p* value		Mean (SD)	Diff. in mean (SD)	Paired t-test *p* value
OH Self- efficacy	Time 0	3.42 (0.55)	− 0.18 (0.59)	< 0.0001	Time 0	3.40 (0.60)	0.01 (0.71)	0.779
	Time 1	3.60 (0.49)			Time 1	3.39 (0.59)		
OH Perceived risk	Time 0	2.95 (0.72)	− 0.11 (0.70)	0.046	Time 0	2.97 (0.76)	− 0.08 (0.86)	0.238
	Time 1	3.06 (0.69)			Time 1	3.05 (0.80)		
OH Fears	Time 0	2.14 (1.03)	− 0.40 (1.0)	< 0.0001	Time 0	2.34 (1.05)	− 0.22 (1.01)	0.008
	Time 1	2.54 (1.08)			Time 1	2.57 (1.12)		
OH Intent	Time 0	1.72 (0.34)	− 0.12 (0.30)	< 0.0001	Time 0	1.62 (0.41)	− 0.08 (0.33)	0.004
	Time 1	1.84 (0.28)			Time 1	1.71 (0.37)		
OH Locus of control	Time 0	2.78 (0.67)	− 0.15 (0.81)	0.014	Time 0	2.82 (0.69)	0.03 (0.74)	0.621
	Time 1	2.94 (0.67)			Time 1	2.78 (0.71)		
OH Norms	Time 0	3.66 (0.42)	− 0.16 (0.53)	< 0.0001	Time 0	3.73 (0.37)	− 0.11 (0.42)	0.002
	Time 1	3.82 (0.35)			Time 1	3.84 (0.28)		
OH worries	Time 0	2.81 (0.86)	− 0.27 (0.70)	< 0.0001	Time 0	2.94 (0.83)	− 0.16 (0.72)	0.007
	Time 1	3.08 (0.78)			Time 1	3.11 (0.84)		
Sugar	Time 0	0.70 (0.49)	0.07 (0.47)	0.052	Time 0	0.71 (0.48)	0.15 (0.49)	< 0.0001
	Time 1	0.63 (0.45)			Time 1	0.56 (0.42)		
Brushing	Time 0	4.02 (.831)	− 0.172	0.001	Time 0	3.88 (.938)	0.021	.683
	Time 1	4.20 (.727)			Time 1	3.86 (.861)		
Flossing	Time 0	2.28 (1.880)	− .718	0.001	Time 0	1.93 (1.75)	− .273	0.029
	Time 1	2.91 (1.534)			Time 1	2.20 (1.730)

## Results

Approximately 70% of residents were 62 years of age and above, and most had incomes typically less than $900.00 a month. About 50% were Hispanic, the remainder predominantly Black (24.7%) and White (9.2%). Around 73.6% were combined Medicare and Medicaid insurance recipients. Medicaid (government insurance based on income) covers basic dental care. Fifty-five percent had been to the dentist in the past year. The majority of those enrolled were living by themselves. Demographics did not differ significantly across the two interventions at baseline (Table 1).

Both GI and plaque score improved significantly from baseline to first follow-up (mean difference .12, *p* > .0001) although the mean differences in improvement were greater in the AMI than in the campaign intervention (see Figs. 2 and 3). While both clinical outcomes improved significantly, the improvement was greater in the AMI than in the campaign intervention group.

Paired T-tests showed that most of the intervention mechanisms improved over time but the amount of improvement in mechanisms differed by intervention. The AMI counseling intervention resulted in improvements in nine domains and the campaign intervention resulted in improvements in three domains. Where the domains improved for both interventions (fears, intent, norms and worries), the mean difference in improvement in the counseling intervention was greater than the mean difference in improvement for the campaign intervention. Three domains improved only in the counseling intervention group, self-efficacy, perceived risk of oral health problems and brushing; one mediator, sugar intake, increased only in the campaign intervention group though the change was trending in the same direction of improvement in the AMI group. Mean differences are shown in Table 2.

Statistical models (GLMMs) were used to examine participant characteristics (moderators) and intervention arm as predictors of each intervention mechanism. Table 3 addresses the question of whether intervention mechanisms change from T0 to T1, and whether the changes are due to time (from pre to post intervention) or the effects of one or the other or both interventions. Four cognitive mechanisms (perceived risk of oral health problems, fear of oral diseases, intentionality, and worries about oral health self-management) and one behavioral mechanism, sugar intake, improved over time across both interventions. Two additional cognitive mechanisms, oral hygiene self-efficacy and oral health locus of control improved in the AMI counseling intervention only. There were no significant changes in brushing and flossing by intervention, meaning that neither intervention made a difference in brushing and flossing when covariates were controlled for. Important participant characteristics associated with intervention mechanisms were education, age, depression, more than one impairing diagnosis, gender and ethnicity. The effect of each of these participant characteristics varied by domain, as noted in Table 3.

**Table 3:** Background characteristics, time and intervention (AMI or campaign) as predictors of intervention mechanisms (GLMM*/GEE**)

	Model		Estimate	Std. error	*p* value	Confidence interval	
						Upper	Lower
1	Self-efficacy	Intervention × time (AMI with Campaign—as ref)	0.199	0.074	0.007	0.053	0.34
		Race (Hispanics vs Blacks—as ref)	0.302	0.066	< .0001	0.17	0.43
2	Perceived risk	Income (More than $900 vs Less than or equal to $900—as ref)	− 0.206	0.071	0.004	− 0.34	− 0.06
		Age (62 and above vs 62 or less—as ref)	0.278	0.074	0.0002	0.13	0.42
		Oral health rating (Good and Excellent vs Poor and Fair—as ref)	0.355	0.069	< .0001	0.21	0.49
		Diagnoses that interfere with daily life activities (1 + vs 0—as ref)	− 0.162	0.072	0.02	− 0.30	− 0.01
		CES-D (4 + vas 0–4—as ref)	− 0.135	0.071	0.05	− 0.27	0.005
3	Fears of oral health diseases	Time (time 1 vs Baseline—as ref)	0.245	0.084	0.003	0.080	0.41
		Age (62 and above vs 62 or less—as ref)	0.293	0.105	0.005	0.085	0.500
		Diagnosis that interfere with daily life activities (1 + vs 0—as ref)	− 0.250	0.102	0.015	− 0.45	− 0.04
		CES-D (4 + vas 0–4—as ref)	− 0.197	0.100	0.051	− 0.39	0.001
		Race (Hispanics vs Blacks—as ref)	− 0.595	0.129	< .0001	− 0.85	− 0.339
4	Oral health intentionality	Time (time 1 vs Baseline—as ref)	0.0752	0.026	0.005	0.022	0.127
		Gender (Female vs Male—as ref)	0.0805	0.034	0.0213	0.012	0.14
		Oral health rating (Good and Excellent vs Poor and Fair—as ref)	0.091	0.035	0.01	0.021	0.16
		Diagnosis that interfere with daily life activities (1 + vs 0—as ref)	− 0.101	0.036	0.006	− 0.173	− 0.028
		Race (White and others vs Blacks—as ref)	− 0.165	0.054	0.002	− 0.271	− 0.05
5	Locus of control	Intervention × time (AMI with Campaign as ref)	0.187	0.090	0.03	0.009	0.36
		Education (Above high school vs Less than high school—as ref)	0.406	0.071	< .0001	0.264	0.54
		Diagnoses that interfere with daily life activities (1 + vs 0—as ref)	− 0.174	0.065	0.008	− 0.304	− 0.04
6	Oral health norm-belief of oral health hygiene and behavior	Intervention (AMI with Campaign—as ref)	− 0.099	0.043	0.02	− 0.186	− 0.013
		Time (1 vs Baseline—as ref)	0.103	0.039	0.008	0.026	0.181
		Oral health rating (Good and Excellent vs Poor and Fair—as ref)	0.061	0.031	0.04	0.0007	0.123
7	Worries of oral health diseases	Time (1 vs Baseline—as ref)	0.18	0.060	0.001	0.069	0.30
		Age (62 and above vs 62 or less—as ref)	0.17	0.084	0.03	0.0081	0.34
		Diagnoses that interfere with daily life activities (1 + vs 0—as ref)	− 0.20	0.082	0.01	− 0.36	− 0.040
		CES-D (4 + vas 0–4—as ref)	− 0.21	0.081	0.007	− 0.37	− 0.057
		Race (Hispanics vs Blacks—as ref)	− 0.45	0.104	< .0001	− 0.66	− 0.25
8	Sugar intake	Time (1 with baseline—as ref)	− 0.2767	0.04396	< .0001	− 0.3632	− 0.1902
9	Brushing frequency	Intervention * time (AMI with Campaign—as ref)	− 0.7117	0.2857	0.0127	− 1.2716	− 0.1519
		Gender (Female vs Male—as ref)	− 0.480	0.226	0.03	− 0.92	− 0.03
		Income (More than $900 vs Less than or equal to $900—as ref)	0.741	0.238	0.001	0.27	1.20
		Diagnosis that interfere with daily life activities (1 + vs 0—as ref)	0.703	0.247	0.004	0.21	1.18
		Race (Hispanics vs Blacks—as ref)	− 0.126	0.289	< .0001	− 2.33	− 1.17
10	Flossing frequency	Intervention * time (AMI with Campaign- as ref)	− 0.5160	0.2455	0.0356	− 0.9972	− 0.0347
		Gender (Female vs Male—as ref)	− 0.567	0.178	0.001	− 0.91	− 0.21
		Diagnoses that interfere with daily life activities (1 + vs 0—as ref)	0.368	0.188	0.05	− 0.001	0.73
		Time	− 0.4216	0.1668	0.0115	− 0.7485	− 0.0946

*This table reports on only significant predictors. Absence of time and condition for a mediator means non-significance

**Models generated with GEE statistic for categorical variables

Statistical models (GLMMs) were used to examine each mediator in relation to each clinical outcome indicate where the variable added a significant mediation effect.

Cognitive mechanism improvements that contributed significantly to declines in GI were locus of control and intentionality. Behavioral mechanisms were tooth brushing and flossing frequency. Decreased sugar consumption had borderline significance. All of these improvements occurred in association with the counseling intervention. Other mechanisms had no influence on GI. Cognitive mechanism improvements that contributed to declines in PS were fears about oral diseases and worries about oral health self-management which occurred in association with both the AMI and the Campaign interventions (Table 4).

**Table 4:** GLMM analysis of intervention mechanisms, time, intervention arm, covariates and clinical outcomes***

Variable	Estimate	Std. error	*p* value	CL	
				Upper	Lower
*Gingival Index*					
Self-efficacy *, **	− 0.02182	0.01692	0.1983	− 0.05512	0.01149
Time	− 0.07759	0.01911	< .0001	− 0.1152	− 0.03998
Intervention*time (AMI)	− 0.07721	0.02646	0.0038	− 0.1293	− 0.02514
Perceived risk *, **	− 0.00143	0.01333	0.9149	− 0.02765	0.02480
Time	− 0.07705	0.01920	< .0001	− 0.1148	− 0.03928
Intervention*time (AMI)	− 0.08163	0.02632	0.0021	− 0.1334	− 0.02984
Intent *, **	− 0.09234	0.03015	0.0024	− 0.1517	− 0.03302
Time	− 0.06996	0.01945	0.0004	− 0.1082	− 0.03169
Intervention*time (AMI)	− 0.07742	0.02656	0.0038	− 0.1297	− 0.02515
Norms *, **	− 0.03425	0.02415	0.1572	− 0.08177	0.01327
Time	− 0.07338	0.01936	0.0002	− 0.1115	− 0.03527
Intervention * time (AMI)	− 0.08009	0.02637	0.0026	− 0.1320	− 0.02821
Locus of control (*)	− 0.04627	0.01366	0.0008	− 0.07314	− 0.01940
Time	− 0.07807	0.01906	< .0001	− 0.1156	− 0.04057
Intervention*time (AMI)	− 0.07298	0.02629	0.0058	− 0.1247	− 0.02125
Worry*, **	− 0.01702	0.01342	0.2056	− 0.04342	0.009383
Time	− 0.07398	0.01932	0.0002	− 0.1120	− 0.03595
Intervention*time (AMI)	− 0.08018	0.02633	0.0025	− 0.1320	− 0.02836
Fears *, **	− 0.01006	0.009907	0.3107	− 0.02956	0.009435
Time	− 0.07467	0.01932	0.0001	− 0.1127	− 0.03666
Intervention * time (AMI)	− 0.08017	0.02636	0.0026	− 0.1320	− 0.02830
Sugar Intake*, **	0.009035	0.01961	0.6454	− 0.02956	0.04763
Time	− 0.07468	0.01992	0.0002	− 0.1139	− 0.03549
Intervention * time (AMI)	− 0.08229	0.02636	0.0020	− 0.1342	− 0.03043
Brushing *, **	− 0.06488	0.02670	0.0157	− 0.1174	− 0.01234
Time	− 0.07580	0.01924	0.0001	− 0.1137	− 0.03794
Intervention * time (AMI)	− 0.07593	0.02652	0.0045	− 0.1281	− 0.02375
Flossing *, **	− 0.05892	0.02018	0.0038	− 0.09863	− 0.01921
Time	− 0.07127	0.01926	0.0003	− 0.1092	− 0.03338
Intervention * time (AMI)	− 0.07495	0.02640	0.0048	− 0.1269	− 0.02300
*Plaque scores*					
Self efficacy*,**	0.003714	0.01318	0.7783	− 0.02222	0.02965
Time	− 0.08234	0.01732	< .0001	− 0.1164	− 0.04825
Intervention *time (AMI)	− 0.08175	0.02391	0.0007	− 0.1288	− 0.03469
Perceived risk *, **	− 0.01705	0.01035	0.1005	− 0.03742	0.003318
Time	− 0.08099	0.01732	< .0001	− 0.1151	− 0.04691
Intervention*time (AMI)	− 0.08052	0.02373	0.0008	− 0.1272	− 0.03383
Intent *, **	− 0.00157	0.02170	0.9422	− 0.04427	0.04112
Time	− 0.08229	0.01739	< .0001	− 0.1165	− 0.04807
Intervention*time (AMI)	− 0.08091	0.02377	0.0008	− 0.1277	− 0.03412
Norms *, **	− 0.02574	0.01887	0.1737	− 0.06287	0.01140
Time	− 0.07957	0.01745	< .0001	− 0.1139	− 0.04523
Intervention * time (AMI)	− 0.07977	0.02378	0.0009	− 0.1266	− 0.03296
Locus of control *, **	− 0.01988	0.01068	0.0638	− 0.04090	0.001148
Time	− 0.08273	0.01731	< .0001	− 0.1168	− 0.04866
Intervention*time (AMI)	− 0.07728	0.02383	0.0013	− 0.1242	− 0.03038
Worry *, **	− 0.02246	0.009910	0.0241	− 0.04197	− 0.00296
Time	− 0.07807	0.01742	< .0001	− 0.1123	− 0.04379
Intervention*time (AMI)	− 0.07914	0.02377	0.0010	− 0.1259	− 0.03236
Fears *,**	− 0.02562	0.007592	0.0008	− 0.04056	− 0.01068
Time	− 0.07602	0.01720	< .0001	− 0.1099	− 0.04217
Intervention*time (AMI)	− 0.07718	0.02348	0.0011	− 0.1234	− 0.03097
Sugar intake *,**	− 0.01819	0.01419	0.2009	− 0.04611	0.009737
Time	− 0.08746	0.01771	< .0001	− 0.1223	− 0.05260
Intervention * time (AMI)	− 0.07973	0.02371	0.0009	− 0.1264	− 0.03307
Brushing *,**	− 0.02395	0.01911	0.2111	− 0.06155	0.01365
Time	− 0.08191	0.01733	< .0001	− 0.1160	− 0.04782
Intervention* time (AMI)	− 0.07882	0.02382	0.0011	− 0.1257	− 0.03194
Flossing *,**	− 0.01959	0.01515	0.1967	− 0.04940	0.01021
Time	− 0.08044	0.01739	< .0001	− 0.1147	− 0.04622
Intervention* time (AMI)	− 0.07872	0.02383	0.0011	− 0.1256	− 0.03182

*Refers to sex as covariate; ** refers to education as covariate ***; Slicing effects show that the change occurs in intervention A versus B across all analyses

In the final GLMM model, (Table 5), declines in GI were predicted by improvements in locus of control and declines in PS were predicted by a decrease in fear of oral diseases. Covariates gender (female) predicted reduced GI score, and female gender plus higher education level predicted reduced plaque scores.

## Discussion

This study was conducted with vulnerable older adults between the ages of 50 and 90 living in subsidized senior housing in central Connecticut who received one of two interventions: a one-hour one-time counseling session tailored to individuals, and a building level campaign consisting of three one and one-half hour oral health fairs. The paper focuses on whether oral hygiene outcomes improve as a result of one or the other intervention; whether mechanisms change as a result of each intervention and whether changes in these mechanisms contribute to clinical outcomes associated with each intervention.

Our primary study hypothesis was that the AMI, a counseling intervention, would have a greater effect on clinical outcomes than the campaign, a group-norms change intervention. Outcomes improved significantly for both interventions but the AMI contributed to better GI and PS scores compared to the Campaign. A second hypothesis was that the AMI would have a greater effect on study psychological and behavioral mechanisms of change than intervention B because it was more intensive and tailored to individual needs when mechanism scores were below the designated cutoff point. This hypothesis was only partially confirmed. As shown in Table 2, the mechanisms reflecting more emotion-related domains (fear of oral diseases and worries), intentionality to act and norms (beliefs about the importance of oral health) improved in both interventions. This is not surprising since both interventions focused on dispelling myths and misunderstandings about oral health fears and worries and emphasized the importance of taking responsibility for oral hygiene behaviors. Brushing improved in the AMI counseling intervention and flossing improved in both interventions but less so in the campaign than in the AMI intervention. An improvement in brushing can be explained by the direct instruction and practice to mastery offered to all participants in the AMI in contrast to demonstration without practice to mastery in the Campaign intervention. An improvement in flossing in both interventions can be explained by the fact that over half of participants at baseline were not flossing properly or at all and/or were unfamiliar with proper flossing techniques. A greater improvement in the AMI than the Campaign was likely attributable to the AMI’s direct instruction to the participant along with feedback on skills practice. Only the AMI intervention accounted for increases in the related mechanisms of self-efficacy and locus of control. This can be explained by the counseling intervention’s focus on gaining control over oral hygiene practices through direct observation and practice to mastery.

The analysis of the relationship between mechanisms and outcomes in our baseline data suggested that improvements in intentionality and locus of control as well as brushing and flossing would result in decreases in Gingival Index. All of these four mechanisms (locus of control, intentionality, brushing and flossing) have been shown in our study to predict GI although only via the AMI counseling intervention. The baseline analysis also predicted that fears and locus of control would be important predictors of PS. Fears has been shown to be a predictor of PS but only via intervention A. Thus, the baseline analyses examining mechanisms in relation to clinical outcomes have, for the most part, been borne out by the results of the first round of intervention especially with respect to the AMI counseling intervention. Surprisingly, no behavioral improvements contributed to declines in PS.

As shown in the trimmed GLMM analysis (Table 5) locus of control was the most important predictor of GI. Locus of control over health behaviors is an important construct in oral health [32, 33], as well as other preventive health behaviors [34], and is related to other predictors of improvement in oral hygiene [35]. The trimmed model shows that reducing fear of oral diseases is an important contributor to flossing. Our results align with other studies showing the importance of emotional factors such as dental anxiety in oral health treatment [36]. Emotional factors are not routinely investigated in oral health preventive behaviors but could provide a new avenue of exploration in future studies. Finally, it is worth noting that though behavioral mechanisms (improved brushing, flossing and sugar intake) were important in the individual GLMM models, none contributed to the final model. In the debate about the relative influence of cognitive and behavioral interventions in oral health, this finding reinforces the importance of including cognitive/emotional components in efforts to improve oral health outcomes.

**Table 5:** GLMM final models with significant intervention mechanisms, intervention, time and outcomes

Effect	Estimate	Standard Error	Pr >|t|	CL	
				Lower	Upper
*Gingival Index*					
Change over time	− 0.063	0.020	0.002	− 0.104	− 0.023
Change in time x intervention (AMI as reference)	− 0.060	0.027	0.026	− 0.114	− 0.007
Gender (Female vs Male—as reference)	− 0.067	0.026	0.012	− 0.120	− 0.015
Locus of control	− 0.037	0.013	0.007	− 0.065	− 0.010
*Plaque Score*					
Change over Time/	− 0.080	0.019	< .0001	− 0.116	− 0.044
Change in Time x Intervention (AMI as reference)	− 0.067	0.024	0.005	− 0.115	− 0.019
Gender (Female vs Male—as reference)	− 0.038	0.017	0.03	− 0.072	− 0.003
Education (More than high school vs Less than high school—as reference)	− 0.089	0.020	< .0001	− 0.130	− 0.049
Fear of oral diseases	− 0.021	0.009	0.026	− 0.040	− 0.002

In terms of covariates, female gender predicted improvements in GI and PS in the final GLMM analysis. More women than men live in senior housing and more women participated in the campaign intervention. Education also contributed to PS. Future interventions with this population should take into consideration both education and health literacy level and make greater efforts to recruit men.

The results suggest that the AMI, a face to face counseling approach, is likely to have a greater impact on clinical outcomes through specific intervention mechanisms than a norms-based oral health campaign with peer facilitation. Though its potential is high, administration has relied on paid professional health educators. With a clearer understanding of which mechanisms are central to improvement and reliance on devices for demonstrating good brushing and flossing practices it may be feasible to train peer educators or health professions students to implement the AMI counseling intervention in community settings such as senior housing apartment buildings or senior centers. The curriculum also is transferrable to community-based clinic settings. At the same time, improvements were also evident in campaign intervention outcomes, and some mechanisms. Though brushing did not increase, flossing did, suggesting that amplifying the demonstration and practice elements of the oral health campaign could produce equivalent results in community settings such as housing projects, senior centers and other places where older adults congregate. Further, the campaign intervention has been shown to be implementable by trained peer educators with professional support.

## Conclusions

This paper had several primary objectives: first to evaluate the utility of the IM model as the basis for establishing and evaluating intervention mechanisms in two interventions driven by the same conceptual framework; second, to illustrate the role of mediators as mechanisms of intervention in relation to clinical outcomes across the two interventions, and finally to provide results that might help interventionists determine which of the two interventions would be most practical and viable in their settings. This paper has demonstrated that both the interventions did improve the psychosocial and behavioral mechanisms guided by the Integrated Model for oral hygiene improvement, and that most of the mechanisms had an effect on the study clinical outcomes. The results also indicate that each of the interventions as implemented works through somewhat different mechanisms. While it appears that the AMI counseling intervention had better overall results, the Campaign intervention also had unexpected positive results suggesting that with the strengthening of the Campaign with the face to face brushing and flossing components of the AMI it can offers an important and innovative alternative intervention approach to introducing oral hygiene into community-based settings.

### Limitations

The study was limited to residents of senior housing in central Ct. But 25% of older low-income adults nationally reside in publicly subsidized senior housing, making the results potentially generalizable. Not all enrolled participants attended the oral health fairs thus possibly contributing to reductions in effect of mechanisms on the counseling intervention. Finally, limitations in the measurement of behavioral mechanisms or the possibility that there could be intervention domains or mechanisms other than those included in the final models might have had an impact on outcomes.

## Data Availability

The datasets generated and/or analyzed during the current study are not publicly available but data used in analyses for the paper are available from the corresponding author on reasonable request.

## Abbreviations

IM: Integrated Model
AMI: Adapted motivational interviewing counseling intervention
PM: Practice to Mastery
GI: Gingival Index
PS: Plaque Score
T0: Time zero—baseline assessment time point
T1: Time 1—post intervention assessment
